# Social Media Usage and Development of Psychiatric Disorders in Childhood and Adolescence: A Review

**DOI:** 10.3389/fpsyt.2020.508595

**Published:** 2021-01-13

**Authors:** Ilaria Cataldo, Bruno Lepri, Michelle Jin Yee Neoh, Gianluca Esposito

**Affiliations:** ^1^Affiliative Behavior and Physiology Lab, Department of Psychology and Cognitive Science, University of Trento, Trento, Italy; ^2^Mobile and Social Computing Lab, Bruno Kessler Foundation, Trento, Italy; ^3^Social and Affective Neuroscience Lab, Psychology Program, School of Social Sciences, Nanyang Technological University, Singapore, Singapore; ^4^Lee Kong Chian School of Medicine, Nanyang Technological University, Singapore, Singapore

**Keywords:** social media, Facebook, Instagram, Twitter, depression, anxiety, adolescence, psychiatric disorders

## Abstract

Social media platforms, such as Facebook, Twitter, and Instagram, are now part of almost everyone's social life, especially for the newer generations. Children and teenagers grew up together with these Internet-based services, which have become an integral part of their personal and social life. However, as reported in various studies, psychological and psychiatric problems are sometimes associated with problematic usage of social media. The primary purpose of this review is to provide an overview of the cognitive, psychological, and social outcomes correlated with a problematic use of social media sites during the developmental stages, from age 10 to 19 years. With a specific focus on depression, anxiety, eating, and neurodevelopmental disorders, the review also discusses evidence related to genetic and neurobiological issues, together with the implications in clinical work and future directions under a multidisciplinary perspective. While the scientific community has made significant progress in enhancing our understanding of the impact of social media on teenagers' lives, more research integrating biological and environmental factors is required to fully elucidate the development of these disorders.

## 1. Social Media: An Increasing Phenomenon in Human Behavior

**Figure 1:**
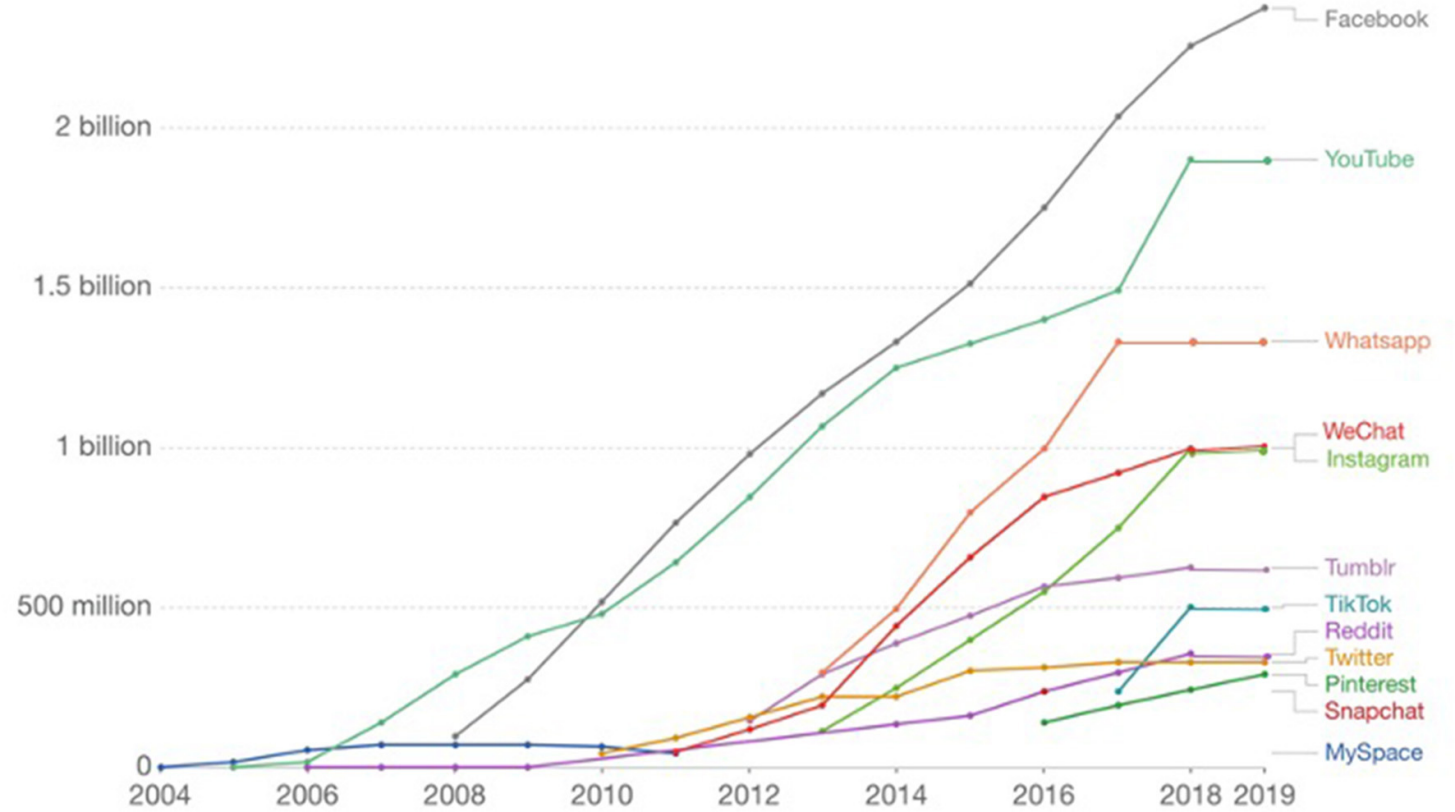
Number of people using social media from 2004 until the end of 2019; estimates correspond to monthly active users, defined as those who logged in during the past 30 days. Source: Esteban Ortiz-Ospina (2019)—“The Rise of Social Media,” published online at ourworldindata.org and retrieved from https://ourworldindata.org/rise-of-social-media.

In our global digital world, social connections are embedded within the external environment we are physically engaged in and the life that we virtually share on social media. Social media is a class of mobile and Internet-based applications that allow people to receive information and to build and share user-generated content. Through the creation of a virtual profile, it is possible to interact with real-life friends, meet new people from all over the world, connect with one's favorite celebrities, and to maintain both online and offline relationships. Since 2004, the use of social media has been increasing rapidly, with the possibility to be connected to the Internet anytime and anywhere. According to the nature of the content, the user can choose, from a wide range of applications, the platform that best suits the purpose of the communication. For example, Facebook is more focused on real-life friends and relatives and encourage interactions through services such as sharing pictures, videos, status updates, and joining groups with specific interests. Social platforms like Twitter, which are also known as “microblogs,” are characterized by brief communication. Other applications, like Instagram or Snapchat, provide photo- and video-sharing services, together with the possibility to like, comment, and re-post preferred content. Figure 1 shows the popularity of the leading social networks, ranked by the worldwide number of active users (source: ourworldindata.org).

Social media platforms are widely used across different age groups and cultures, but especially for children and teenagers, online communication represents “*a window into the secret world of adolescent peer culture, even as it offers young people a new screen for the projection of adolescent developmental issues*” (1). While social media offers tremendous potential in allowing self-expression of personality and maintaining contact with a network of friends, some studies have also highlighted the risk of negative consequences of excessive online social platforms usage (2, 3). Online social interaction, the blurring of lines between offline and virtual life (4, 5), and the concept of digital identity (6) have become topics of great interest in psychology and mental health fields (7). Researchers in the field are attempting to find a consensual definition of the concept of “problematic social media use,” as it is often confused with a description of addictive behavior related to general Internet services, which has been included in the 5th edition of the Diagnostic and Statistical Manual of mental disorders (8). In accordance with a biopsychosocial framework, problematic use of social media involves a set of alterations affecting biological functions (i.e., neurotransmitters regulation and circadian rhythm); cognitive, psychological, and affective mechanisms (i.e., attention, salience, mood fluctuation, and anxiety), and aspects related to the social sphere (i.e., social desirability, popularity, and conflicts), resulting in a decreased perceived quality of life. Feedback from people belonging to the virtual social community can affect individual self-esteem and, generally, well-being (9–13). A problematic use can also affect other aspects of a teenager's daily life, such as academic performance, time management issues, procrastination, distraction (14), and sleep disturbances (15). In severe cases, averse outcomes could arise and, if prolonged, can become highly impactful, with the further risk of developing psychiatric disorders (16). As the Internet and social media are a recent phenomenon, it is more likely that the effect of excessive or problematic usage will affect individuals during more sensitive temporal frames, such as childhood and adolescence. A survey conducted in the United States in 2018 reported that 45% of the teenagers interviewed say they are almost constantly online, without differences among sexes, ethnicities, family incomes, and parental level of education (for the full report, see Teens, Social Media & Technology 2018). Given the continuous exposure to the virtual environment, it is essential to understand the impact that online social relationships have on mental health and interpersonal functioning in developmental stages. The aim of our review, compared to other recent publications [see (17, 18)], is to provide a detailed overview of not only the effect of social media in general but also of the associations between specific platforms and psychopathology. We believe that this point is relevant, as it is important to distinguish among the different social media platforms given that each of them has specific, unique features that drive young users' preferences. Furthermore, social media usage is often included in the broader category of Internet usage, despite the social connotation that primarily describes and defines these kinds of sites. Moreover, the included articles were discussed according to specific disorders that can develop during childhood and adolescence, not merely depression and anxiety that are the most explored disorders but also addictive behaviors toward substances and eating disorders (EDs), as both start to develop during adolescence. In fact, developmental stages are more vulnerable to environmental insults just because of the greater plasticity of the central nervous system, the multiple biological changes, and the formation of psychological mechanisms that drive social behaviors (19, 20). Due to the differences that define each platform, one of the main purposes of the present review is to provide evidence related to targeted social media services, instead of a more general discussion on social media. In fact, we retain that the multifaceted manifestation of diverse psychological issues might be expressed differently through the multiple ways of communication, such as text, video, or picture. As social behavior and the risk for psychiatric disorders is related to the activity of determined brain regions and biological features (21, 22), and since we are addressing the outcomes of problematic social media usage (PSMU) under a biopsychosocial perspective, we will also provide an overview about the neuroscientific and gene-by-environment contribution to the interplay between social media and the development of psychiatric disorders in adolescence.

## 2. Methods and Results

The review adopted the Preferred Reporting Items for Systematic reviews and Meta-Analyses (PRISMA) model in conducting a systematic literature review. A search of four scientific electronic databases yielded 42 papers for qualitative evaluation. We searched PubMed Central, PubMed, PsycInfo, and Scopus databases for articles on psychiatric disorders in youths related to social media. Since this topic embraces multiple fields, such as computer science and information and communication technologies, we also browsed the Association for Computing Machinery Digital Library and the Institute of Electrical and Electronics Engineers Xplore Digital Library to find relevant research articles in the proceedings of conferences focused on the role of social media in explaining psychological issues in the developmental age. We comparatively analyzed the literature from 2006 up to the end of July 2020, combining different keywords and Boolean operators. A database was generated by combining terms and Boolean operators, such as “social media” AND “child*,” “social media use” AND “child*,” “social media” AND “disorder” AND “youth*”. To include more targeted records, we conducted a further search on the same databases using terms describing the specific issues we meant to address in this review: (“YouTube” OR “WeChat” OR “TikTok” OR “Reddit” OR “Pinterest” OR “Facebook” OR “Instagram” OR “Twitter” OR “Tumblr” OR “MySpace” OR “Whatsapp”) AND (“psychiatric disorder” OR “mental health” OR “psychological well-being”) AND (“adolescent*” OR “youth*” OR “teenager*”).

### 2.1. Eligibility Criteria

From a methodological perspective, studies had to fulfill the following criteria to be included: journals and proceedings of conference papers published up to the end of July 2020, published in English, and meeting the following criteria:

1. participants: children and adolescents until the age of 19 with a profile on at least one of the most popular social media platforms (Facebook, YouTube, WhatsApp, WeChat, Instagram, Twitter, TikTok, Tumblr, Reddit, Pinterest, Snapchat, MySpace, Q-Zone); we opted to consider the age of 19 as the upper limit of adolescence, in accordance with the definition provided by the World Health Organization https://apps.who.int/adolescent/second-decade/section2/page1/recognizing-adolescence.html;
2. interventions: assessment of psychiatric disorders in the developmental ages (depressive symptoms, anxiety and related issues, EDs and body dissatisfaction, neurodevelopmental disorders, substance misuse or abuse);
3. comparison: it is not applicable, as we only included studies based on the sample of social media users;
4. outcomes: we considered the levels of psychological well-being or diagnosis of psychiatric disorders as the outcome;
5. study design: we included studies containing quantitative approaches to produce empirical data and qualitative designs.

### 2.2. Results

**Figure 2:**
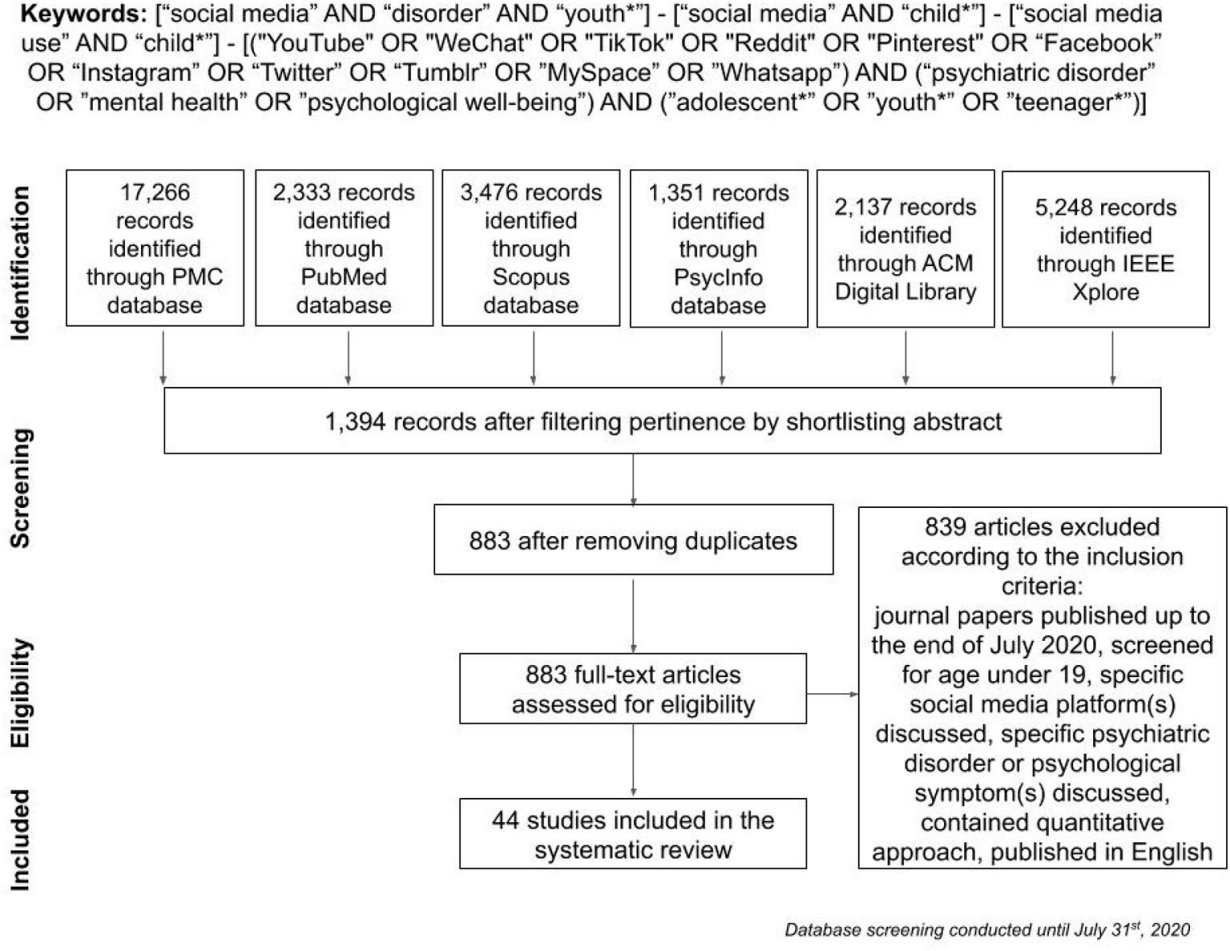
Flow chart of procedural articles shortlisting according to the Preferred Reporting Items for Systematic reviews and Meta-Analyses (PRISMA) guidelines.

For the selection procedure for the included articles, please refer to Figure 2. In the results, we will discuss only the studies resulting from the literature research. In Table 1, all the articles included in the review are listed, together with the principal information. Effect size computations for each study have been performed using an effect size calculator (64) or calculated manually. When more variables were analyzed in the study, we reported the range of values for effect sizes (Cohen's *d*). Disorders will be discussed in distinguished macro-categories, divided by diagnostic class, according to the DSM-5(65). Relevant topics such as involvement/changes of neural correlates and genetic contribution will also be discussed. A total of 31,823 papers were screened by title and abstract, 1,394 were considered for further screening, and 511 duplicate papers were removed. Note that 839 papers were removed after assessment for eligibility according to the exclusion criteria, resulting in 44 papers included in the review.

**Table 1:** List of the studies included in the review.

**n**	**Article**	**Age**	***N***	**Social media**	**Disorder/symptoms**	**Findings**	***ES***
1	Szwedo et al. (23)	13/20	89	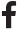 , MS	Depressive symptoms, social anxiety	– (depr); + (s.anx)	0.40–0.60
2	Moreno et al. (24)	18–19	66	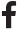	Alcohol use	+	0.72
3	Pumper and Moreno (25)	12–14	315	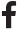	Alcohol use	+	0.15
4	Tiggemann and Slater (26)	13–15	1,087	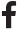 , MS	Body image concerns	+	0.26
5	D'Angelo et al. (27)	18–19	312	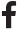	Alcohol use	+	0.20
6	Huang et al. (28)	14–15	1,563	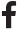 , MS	Alcohol and cigarette use	ns	na
7	Birnbaum et al. (29))	12–21	80	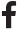 , 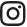 , 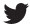	Psycotic-spectrum and mood disorder	na	na
8	Nesi and Prinstein (30)	12–16	619	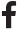 , 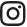	Depressive symptoms	+	0.53
9	Bert et al. (31)	18	341	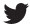	Pro-anorexia	na	na
10	Ehrenreich and Underwood (2016)	18	125	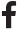	Internalizing symptoms	+	0.58
11	Frison et al. (32)	12–19	1,612	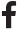	Depressive symptoms	ns	0.58
12	Marczinski et al. (33)	19	146	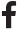	Alcohol use	+	0.44
13	Moreno et al. (34)	17–19	94	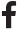 , 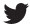	Alcohol use	+	0.47–0.92
14	Naeemi and Tamam (35)	13–16	401	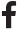	Psychological well-being	–	0.67
15	Sampasa-Kanyinga and Chaput (36)	11–19	4,468	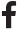 , 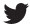 , 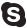 , 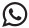	Body image concerns	+	0.39
16	Abar et al. (37)	19	252	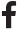	Substance use	na	na
17	Frison and Eggermont (38)	12–19	671	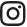	Depressed mood	+	0.42
18	Gul et al. (39)	13–19	289	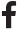	ADHD	+	0.69
19	Jacob et al. (40)	16–24	21	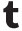	Self-injury	+	na
20	Nesi et al. (30)	15–16	658	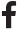	Alcohol use	+	0.43
21	Nesi et al. (30)	13–16	816	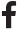 , 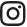	Depressive symptoms	+	0.85
22	Pontes (41)	10-18	509	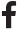	Depressive symptoms, anxiety	+	0.62–0.68
23	Spilkova et al. (42)	16	4,887	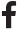 , 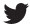 , 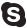 , 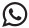	Binge drinking, marjiuana use	+ (drink); ns (marj)	0.88
24	van Rooij et al. (43)	12–15	3,945	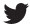	Depressive symptoms, social anxiety	+	0.45–0.95
25	Weinstein (44)	14–18	507	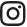	Depressive symptoms	+	0.68
26	Brown et al. (45)	16	52	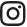	Self-injury, suicidal ideation	na	na
27	Muzaffar et al. (46)	12–20	102	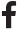	Depressive symptoms, social anxiety	ns	na
28	Niu et al. (47)	12–18	764	QZ	Depressive symptoms	+	0.44
29	Settanni et al. (48)	15	283	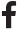	ADHD symptoms	+	0.56
30	Chang et al. (49)	12–16	303	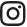	Body esteem	–	0.58
31	de Vries et al. (50)	12–19	440	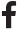	Body dissatisfaction	+	0.49
32	Louragli et al. (51)	12–19	541	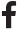	Anxiety, nomophobia	+	0.50–0.98
33	Negriff (52)	13/21	319	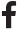	Depressive symptoms	-	0.58
34	Przepiorka and Blachnio (53)	12–17	426	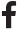	Depressive symptoms	+	0.83
35	Raudsepp and Kais (54)	13	397	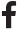 , 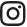 , 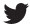	Depressive symptoms	+	0.72
36	Savolainen et al. (55)	15–25	4,816	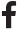 , 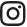 , 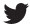 , 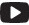	Alcohol use	+	0.12–0.43
37	Shakir et al. (2019)	12–18	537	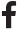 , 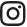 , 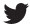 , 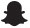 , 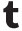	Cyberbullying	na	na
38	Steers et al. (56)	17–19	316	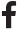	alcohol use	+	0.69
39	Vannucci and Ohannessian (57)	11-14	1205	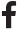 , 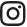 , 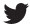 , 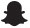 , 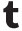	Depressive symptoms, panic disorder symptoms	+	0.28-.92
40	Yurdagül et al. (58)	14–19	491	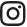	Depressive symptoms, anxiety, social anxiety, body dissatisfaction	+	0.28–0.50
41	Boursier et al. (59)	13–19	693	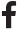 , 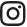 , 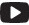 , 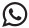	Body image concerns	+	0.36
42	Fardouly et al. (60)	10–12	528	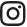 , 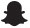 , 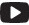	Depressive symptoms, social anxiety, body satisfaction	+ (depr.); ns (s.anx); – (body)	0.43–0.82
43	Stockdale and Coyne (61)	17/19	385	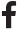 , 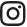 , 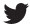	Depressive symptoms, anxiety	ns (depr.); + (anx)	0.36
44	Brown et al. (62)	16	59	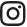	Self-injury	na	na

*Age, range of age of the participants; N, sample size; ES, effect size; +, directly proportional; –, inversely proportional; ns, non-significant; na, not applicable; 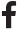, Facebook; 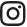, Instagram; 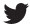, Twitter; 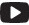, YouTube; 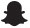, Snapchat; 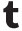, Tumblr; 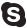, Skype; MS, MySpace; QZ, QZone. Icons of social media platforms have been created using the fontawesome package (63)*.

### 2.3. Depressive Symptoms and Mood Disorders

Depression is a prevalent mood disorder, in which symptoms include persistent sadness and a loss of interest in activities that the person enjoys typically, together with the inability to carry out daily activities (65). With regard to childhood and adolescence, interpersonal models of depression in developmental ages accentuate the cyclical associations between social experiences and depressive symptoms. New schemes in the interpersonal environment, with more articulated, frequent, and unsupervised contacts, may represent a further complication as the influence of peer relationships may affect a person's identity and psychological well-being (66). As depression and internalizing symptoms have increased among youths in the last decade (67), it is vital to question to what extent social media usage is directly linked to this and to understand how they impact each other.

#### 2.3.1. Effects of Social Media Usage on Depressive Symptoms

Given that social media provide users with a range of possible activities, it is possible to identify specific patterns of usage. For instance, a set of actions such as browsing other users' photos or scrolling through comments or news feeds has been labeled as passive social media use. Recent research indicates that this sort of behavior and depression are linked in both directions. Passive social media usage could directly aggravate depressive symptoms, like loss of interest or blue mood, and thwart personal well-being (16, 32, 68, 69). High social media use appears to be predictive of depressive symptoms and low offline social support from both family and peers (57). It might also act indirectly through mediators such as reduced sense of belonging (70), hence increasing levels of loneliness first (43) and, subsequently, depressive mood and stress (16), which, in turn, reinforce each other (68).

#### 2.3.2. Effects of Depressive Symptoms on Social Media Usage

Passive social media use appears to be increased by depressive symptoms, loneliness, and high levels of stress. In a longitudinal study, Kross and colleagues have demonstrated that a sense of loneliness is a predictor for more intense usage of social media (71), as it might represent a solution to alleviate depressed mood, reinforcing PSMU (68). Specific kinds of actions on social media, related to the peculiarity of the site, were found to be associated with adverse emotional and relational outcomes at different times and vice versa. With regard to Instagram, Frison and Eggermont reported that former browsing behavior was related to a later increase in depressed mood (38). Moreover, levels of depressed mood at Time 1 were associated with increased Instagram posting at Time 2, without differences between boys and girls (38). As for Facebook, levels of depressive symptoms at the first stage can be predictive of a lower number of Facebook friends and fewer ties between friends in the second stage (52). Another study based on Facebook data highlights the relationships between internalizing symptoms and online communication in terms of received comments offering support in response to posts indicating negative or depressive emotional states, with girls receiving more backing compared to boys. Such rumination-like behavior through social media might affect negatively not only the mood of the person who posts but also of those who respond, increasing levels of internalizing symptoms and depression (72). Depressive symptoms, together with sleep problems, can represent a positive predictor for excessive involvement in Facebook-related activities (53). Similarly, emotional dependence on Facebook has been found to be negatively correlated to several aspects of adolescents' psychological well-being, such as autonomy, purpose in life, positive relationships, personal growth, self-acceptance, and ability to manage one's environment (35). An addictive attitude toward Facebook was found to be positively correlated with depression, regardless of age (age range 10–18) and gender (41). Longitudinal research on adolescent girls found an association between changes in PSMU and changes in depressive symptoms in both directions, with baseline levels of depressive symptoms being predictive of PSMU (54).

#### 2.3.3. Social Comparison and Negative Affect

Social comparison is a mechanism highly involved in the development of a person's identity starting from childhood, where evaluations are more distorted especially in a positive way, throughout adolescence, when the greater development of cognitive skills permit the generation of more realistic estimates (73). Social comparison, as a consequence, can generate both a positive or a negative self-appraisal, affecting the way people, especially teenagers, perceive themselves and their quality of life. Evidence in literature suggests that PSMU and depressive symptoms might be mediated by social comparisons with others' lives as they appear on their profiles (44, 47, 66), generating a sense of inferiority and feelings of worthlessness (74–78). As a consequence, people showing downward social comparisons are more likely to seek offline feedback for reassurance (66). Social comparison is closely linked to self-esteem, which, in turn, resents of the effect of individual cognitive appraisal, acting as a moderator in the processing of comparison. As a consequence, lower levels of self-esteem can represent a risk factor when making comparisons with others' lives (47). These results appear to be more evident in girls, compared to boys, (44, 66) suggesting that intrinsic features of female identity development can represent a vulnerability for a more negative self-appraisal, especially when comparing or evaluating physical features or attractiveness (49, 54). Moreover, it is possible that online parasocial relationships may amplify distorted perceptions, due to the filtered and selective nature of the information shared, principally when evaluating profiles of users that do not belong to a close or offline network (44).

#### 2.3.4. Controversial Results in the Association Between Depressive Symptoms and Social Media Usage

Amid the research investigating the connection between social media usage and depressive symptoms, a few studies reported no evidence linking social media sites and depression. A recent study investigated the relationships between reasons for Facebook use and psychological and mental health outcomes for a 3-year period in late adolescents, aged from 17 to 19 years. According to their results, none of the possible motivations, which were social connection, boredom, and information seeking, were correlated to depression at any stage of the experimental procedure (61). As for the short-term consequences of negative experiences on Facebook, online peer victimization is not predictive of increased depressive symptoms after 6 months (79). In addition, Fardouly and colleagues did not find differences between users and non-users of the most popular social media platforms (Youtube, Instagram, and Snapchat) among Australian preadolescents in terms of depressive symptoms. Taken together, these results suggest that low mood derived from social media usage might be explained through different factors, such as worry about how youths appear on their preferred social networks sites and their tendency to compare their own image to someone else's image (60). Finally, a longitudinal study by Szwedo and colleagues investigated the preference for Facebook and/or MySpace communication in a cohort of adolescents in relation to depressive symptoms, assessing the sample at the age of 13 (Time 1) and 20 (Time 2). Interestingly, higher depressive symptoms at Time 1 predicted a preference for communication via social media, but at Time 2, higher depressive symptoms were predictive of lesser online disclosure (23). This change in direction might be explained by the different ways, especially social withdrawal, through which depression is manifested in early adolescence and early adulthood. With regard to psychotic and non-psychotic mood disorders, social platforms such as Facebook and Twitter represent an initial avenue to seek help by diagnosed youths (29) and a potential base to examine depressive symptoms and perceived social support from online friends (80).

### 2.4. Anxiety Disorders

Symptoms relating to anxiety often overlap with depression, especially in youths; just like depression, anxious manifestations may result from a set of internal and external circumstances. In social media, where the relational component is strong, anxiety can derive from a perception of being connected inappropriately, from negative online peer-comparison, or from reduced emotion-regulation abilities, as online interaction can be used as a surrogate for offline physical interaction (81). Targeted Facebook features, such as seeking online approval and support through the number of "likes," or only retaining the visibility of posts and pictures that received lots of positive feedback on one's profile, can promote or elicit non-adaptive behaviors (i.e., excessive social comparison and rumination) and increase anxiety-related traits, such as socially prescribed perfectionism, aggravating pre-existing symptoms in youths diagnosed with an anxiety disorder (82). Facebook can also be used by teenagers as a pastime when feeling bored: a 3-year study found that usage of Facebook in order to alleviate boredom at stage 1 (17 years old) was correlated with increased levels of anxiety at a following stage (19 years old), indicating that the anxiety might be a secondary product of the problematic use of social media developed over the two time-points (61). This could reflect the fact that a 3-year window frame can encompass different stages of a teenager's life, especially when approaching emerging adulthood. As the high school period is over, fewer amounts of structured time, coupled with less monitoring behavior by parents and teachers and greater accessibility to smartphones or other electronic devices, can result in an increase in problematic usage of social media and, as a consequence, underlying anxiety-related mechanisms (61). The type and the reiteration of a set of behaviors that Facebook users could engage in (e.g., posting a photo/comment/status update, “liking" behavior, or using the instant message) can be linked with levels of general anxiety. This might be explained by the need to keep worries related to that driving the person to frequently check a previous posting behavior (46). With regards to Instagram, which is more focused on visual contents, one study reported a direct association between Instagram usage with general anxiety in boys, while in girls this link was mediated by body image dissatisfaction, leading to different adverse outcomes in the two groups (58). This difference between genders suggests that females might be more prone to engage in social comparison, especially when it involves physical appearance. This might be because their perception of their ideal body image as being thin is affected by their excessive exposure to attractive celebrity and peer images on Instagram. Moreover, it underlines once again the importance of considering the possible concurrent mechanisms that contribute to the development of psychological issues.

#### 2.4.1. Online Social Anxiety

Social anxiety is described by the enduring preoccupation of being judged negatively by others during a social performance or social circumstances (65). The worry of receiving unfavorable feedback is even stronger during adolescence, when the identity of the self is developing. Online activity on social media can be very attractive, especially for young people with such fears, as it is possible to share information or content in a more controllable environment. Although this allows people with social anxiety issues to overcome, even partially, the fear of being exposed to public judgment, it can lead to the development of a problematic usage of social media platforms. With regard to Facebook, a longitudinal study by Szwedo and colleagues found that at 13 years of age (Time 1), social anxiety does not explain preference for virtual communications, and at 20 years of age (Time 2), it was positively correlated with a predilection for online relations, especially for those expressing increased levels of maternal behavior undermining autonomy at Time 1 (23). Levels of social anxiety in social media young users have been shown to be positively correlated with online behavioral dimensions such as the attitude of comparing one's appearance with other people's pictures on YouTube, Instagram, and Snapchat (60). As a consequence, the approach toward social media can be conflicting: the person desires at the same time to be recognized as interesting and “liked,” but would also like to avoid being judged negatively or ridiculed. The awareness of these mechanisms might intensify pre-existing symptoms of social anxiety, leading to non-adaptive patterns of behavior (82).

#### 2.4.2. Fear of Missing Out and Nomophobia: The Urge to Be Constantly Online

The more people share their lives on their online profiles, the more they are at risk of being afraid of missing updates and feeling the urge to check their profiles for feedback (16, 83). This specific phenomenon has been labeled “fear of missing out" (FoMO), defining the pervasive anxiety experienced by a user when thinking that other people might be enjoying gratifying experiences in their physical absence, pushing him/her to be connected constantly to check upon updates about these experiences, hence fostering the addictive behavior circuit (16, 84–86). FoMO has been shown to be associated with the severity of Facebook usage through a process that is likely to be activated by users as a way to temporarily compensate or regulate negative affect and anxious manifestations (87). Specific social needs may underlie FoMO and reasons for social media usage, like the desire to be popular, or at least not unpopular in the eyes of peers and the need for social affiliation, especially during adolescence when peers acquire greater value compared to the family (88). To this purpose, online interaction can represent a constantly available means of gratification but, at the same time, an attractive risk as it might trigger addictive behaviors and aggravate symptoms of anxiety. This combination of behavioral and cognitive patterns, in the context of social media usage, appears to be mediated by nomophobia, which is described as the fear of not being able to use the mobile phone. Evidence in literature reports a direct association among levels of anxiety, addictive behavior toward social media (41) and nomophobia, with a negative impact on academic performances (51).

### 2.5. Feeding, Eating Disorders, and Body Dissatisfaction

Adolescence is a temporal frame during which physical changes and identity development occur, and teenagers acquire a greater awareness of the body, both their own and those of their peers (49). Posting pictures on social media is one of the most common practices among young people, especially self-photos (commonly known as “selfies”) (89). Exposing and being exposed massively to pictures of body might lead to negative outcomes, such as body image dissatisfaction, defined as “*the discrepancy between identification of one's own figure (actual) and the figure chosen as the desirable self-image*” (90), or alterations in nutrition habits, to the extent of the development of EDs. With regard to Instagram, body image dissatisfaction mediates the relationship between PSMU and internalizing symptoms differently in males and females, with the latter showing a stronger indirect effect (58). Evidence from a study involving Singaporean girls showed that selfie practice on Instagram (browsing and editing) and body esteem are mediated by appearance comparison operated by peers' groups with a negative association, while posting self photos and body esteem are directly correlated (49). With regard to Facebook, Tiggemann and colleagues investigated social media exposure and body image concerns in girls, finding that time spent on the online platform was strongly correlated to body surveillance and the ideal of a thin body shape (26). An analysis of a Canadian sample of teenagers highlights that more frequent and prolonged usage of social media services is associated with body dissatisfaction, with a trend to perceive oneself overweight in both boys and girls (36). Recent findings from a study by Fardouly and colleagues indicate that more frequent appearance comparisons with others on social media and considering them to be more attractive than oneself is negatively correlated with body image satisfaction and positively linked with eating-related disorders in both male and female teenagers (60). Evidence from a sample of Italian adolescents highlights the role played by appearance control beliefs and body image control in photos, as these dimensions could be configured as predictors of problematic usage of social media and negative mental health outcomes (59). Overall, the findings indicate a higher vulnerability for girls to develop a negative image of their own body. This risk can be compounded by misleading and harmful content that can be found on social media.

#### 2.5.1. Presentation of Eating Disorders on Social Media Platforms

In recent years, groups supporting anorexia nervosa in several ways (endorsement and promotion of dysfunctional eating behavior, maintenance of the disorder, and interference with recovery) have been spreading across social media platforms. The dynamics of the Proana Movement, which promotes behaviors relating to anorexia nervosa, have been examined using Twitter, finding that adhering people and/or promoters were almost totally teenage girls (31). In the midst of the factors mediating the risk to develop body dissatisfaction or EDs, one study focused on teenagers' offline social environment, finding that a positive mother-adolescent relationship can exert a protective function against the adverse effects of social media usage on body perception (50). An alarming factor is determined by the support of pro-EDs in online networks. As popular platforms started blocking pro-ED related terms, users supporting dangerous eating habits have begun altering the hashtags, bringing forward their approval toward endangering conducts. On the other hand, it is not unusual that people rehabilitating from an ED seek support during their journey to recovery by sharing their testimony through textual posts or visual media (i.e., pictures, video, and gif). This dual nature of online communication represents a great challenge for research, as the analysis focused uniquely on hashtags may be misleading (91). Moreover, people supporting ED behaviors often alter the terms in hashtags or post them in comments in order to overcome social media censorship policy, with a possible risk to expose more fragile or sensitive people to explicit content.

### 2.6. Alcohol Use/Abuse and Addiction

Adolescence is the stage of life where people gain more independence and make new experiences in their social environment, where peer influence might encourage and provide opportunities to come into contact with alcohol, potentially leading to the development of an addictive behavior toward the substance. As the social environment is now composed of two realities, online and offline, it is crucial to understand the contribution of social media in fostering, maintaining, or conveying contents related to substances. Studies on drinking behavior among teenagers and social media use highlight that online platforms like Facebook might represent a helpful tool to detect problematic alcohol use (25, 33, 34, 92, 93), or advertise for healthy behavior in settings such as popular alcohol-related events and parties (24). A higher number of alcohol-related posts has been shown to be linked to greater drinking conduct and approval from friends, although heavier consumers seem to tend to post less over time compared to light drinkers (56). A longitudinal study revealed that in the Facebook profiles of individuals identified as dependent alcohol users, alcohol references increased and half of those identified referenced intoxication or problematic drinking after 1 year (25), while another longitudinal study indicated that alcohol references at a first stage can predict binge drinking later in time (27). With regard to alcohol-related attitudes, binge drinkers appear to be more prone to use social media excessively (42). Moreover, posts containing references to alcohol predict the number of weekly substance consumption (93), the risk of developing an addiction, and alcohol cravings (92). In order to predict drinking conduct, Marczinski and colleagues have developed the Alcohol-Related Facebook Activity (ARFA) questionnaire (33) based on a sample of college students. The preference for the virtual environment as a platform to share alcohol-related experiences has been studied by Moreno and colleagues, who report that students owning a profile on both Facebook and Twitter tended to post more alcohol references on Facebook compared to Twitter (34), as they were entertaining more social connections on the former site. Online social networks often include connections with offline friends; therefore, the exposure to a friend's drinking pictures or posts can be associated with higher alcohol consumption (28, 30). Risky alcohol behavior can differ according to the country; a cross-cultural study examined the relationship between daily usage of popular social media platforms and alcohol consumption among youths in the United States, Spain, Finland, and South Korea. In the targeted countries, the different platforms were correlated with greater hazardous alcohol usage as follows: Facebook and Instagram in Spain, Finland, and South Korea, YouTube in South Korea, and Twitter in Spain (55). These results suggest that specific social media sites might play an attractive or inspiring role in risky alcohol consumption but, on the other hand, they could also turn out to contribute greatly to online-based interventions. According to a study on nicotine, alcohol, and marijuana consumption in high school, being friends on Facebook with one's own parents and not hiding contents can represent a protective factor against substance use (37). Parental inclusion on social media interactions, without undermining autonomy and privacy of youths, can depict an important element in substance use prevention targeted toward youths.

### 2.7. Self-Harm and Suicidal Ideation

Amid the psychological issues potentially occurring in young people, self-harm is a primary concern, with harmful behaviors lying on a continuum between non-suicidal self-injury (NSSI) and suicidal intention (40). Social media can influence self-injury tendencies negatively, through fostering conducts, contagion, or competitions (94), but they can also represent the first foothold when support is needed. A study based on the analysis of MySpace profiles indicates that teenagers utilize personal virtual space to share their suicidal ideation and behaviors directly or by reporting desperation, hopelessness, and despair (95). From the interviews with adolescents recently collected by Jacob and colleagues about self-harm behaviors, it emerges that Tumblr is the preferred platform to share self-injuring content, like pictures, in an anonymous way, with the consequent risk to normalize such harmful behaviors (40). Looking into the motives that push young people to share self-injury related content such as their own wounds on Instagram, there are mostly social purposes, like the need to belong to a group where the person can feel understood (62). Another reason might be the need to self-disclose in an environment that can guarantee anonymity. These reasons are reported to be valid both for the first NSSI post and for the general NSSI ones. Beyond self-oriented motives, another aim is to raise awareness about the topic in order to help other people (62). Although results concerning Instagram do not report any risk for acute suicidality (96), photos of self-injury practices might play a reinforcement role as they are often posted (45) and frequently concealed behind ambiguous hashtags (97). In fact, as users often resort to the use of hashtags to track the shared contents and to find images or discussions related to specific topics, those regarding self-harming behaviors can contain non-related words (i.e., “blithe” for self-cutting pictures) or be constantly changed, in order to make them easily accessible only to a restricted community (98). Social media-related suicidal behavior is a topic of increasing interest and critical importance that has garnered the attention of newspapers and newscasts all over the world, concerning popular and unpopular people (see Channel News Asia for a recent episode). Although researchers attempted to study the extent of social media on suicidal behaviors in-depth, complexities derive from legal and privacy issues, as well as from the indirect association between the usage on web-based platforms and the suicide itself (99).

### 2.8. Cyberbullying

Suicidal ideation can also derive from the non-adaptive usage of online communication by others, as in the case of cyberbullying. Cyberbullying can be defined as the intentional use of information and communication technologies such as electronic mail, smartphone, short message services, and social media platforms, carried out repeatedly by a group or an individual, to support deliberate, repeated, and hostile behaviors against a victim who cannot easily defend him- or herself (100, 101). Cyberbullying constitutes a possible worrisome phenomenon, given its devastating, occasionally even fatal, consequences on a person's life. Recent statistics point out that cyberbullying is prevalent on platforms based on visual content, such as Instagram (42%), Facebook (37%), and Snapchat (31%) (see the article by Petrov C. on statistics about cyberbullying, February 28, 2019). As the contents are shared and spread quickly online, the victim can experience, besides a lack of control, a series of highly negative psychological consequences, such as social anxiety (102), depression, and suicidal ideation and attempt, especially when bullying behavior perpetuates across time (103, 104). An investigation on social media usage and youths' mental health revealed that cyberbullying appears to mediate this relation occurring in a set of negative outcomes, such as sleep problems and anxiety, more than the frequency of exposure to social media itself, with girls being more exposed to these effects (10). However, social media started adding certain features including the ability to report inappropriate content, comments, and to block users in order to stem violent and inappropriate behaviors.

#### 2.8.1. Safety Measures Adopted by Social Media Sites

Initially, the different platforms did not take responsibility for single users' online behaviors. However, the growing prevalence of cyberbullying in recent years has gained increased relevance, resulting in the implementation of several measures aimed at both children and parents. For instance, in 2013, Facebook launched a safety section on its site, providing information on policies, tools to increase profile protection, and relevant resources and contacts to access in the case of cyber abuse (see Facebook Safety page). Likewise, in 2015, Twitter activated a safety center for parents and teens with guidelines for a more secure navigation and utilization of the site. Furthermore, they founded the Twitter Trust and Safety Council that works in partnership with several institutions and organizations in order to direct users to the appropriate service in case of abuse (see Twitter Safety Partner). With regard to Instagram, which has been owned by Facebook since 2012, the platform presents the community guidelines and another section where parents can find more information about the accessibility and visibility of their children by other users. Moreover, an online form is available for reporting self-injury material, hate comments, abusive or inappropriate content, and profiles belonging to teens younger than 13 years old, which is the requirement to own a profile (see Instagram Privacy and Safety Center page). The same subscription criteria are applied to YouTube, although videos posted by other users are accessible even without owning a profile. Because of this, it is possible for parents to set restrictions in order to avoid potentially dangerous or improper material. In addition, together with the site policies, informative material about harmful behaviors such as self-injury, suicide, harassment, and cyberbullying is provided (see YouTube Community Guidelines). So far, statistics about the efficacy of these safety measures have not being available. Generally, targeted services for prevention have been made known on the most popular online platforms by providing users with links to websites, hotlines, and information about how to detect warning signs of suicide. Web communities focused on suicide prevention have been founded, giving their members the opportunity to share their own direct or indirect experience in an anonymous way and to support each other, without the constraints of physical boundaries (99).

### 2.9. Neurodevelopmental Disorders

Neurodevelopmental disorders are characterized by altered functioning of the neurological system and brain, affecting cognitive functions and social behavior. Although social media interfere with offline interaction by reducing the investment of time and resources in them while offering a more immediate alternative to satisfy social needs, they can also simplify the engagement in social contacts. This feature might be suitable, for instance, for youths with autism spectrum disorders, as they can have difficulties in decoding complex social information (105, 106). As adolescence is a crucial developmental stage where interactions with peers occur both online and offline, it is of pivotal relevance to understand the impact of social media platforms on teenagers with neurodevelopmental disorders. With regard to ASD, evidence shows a positive association between Facebook usage and friendship quality, moderated by anxiety levels, suggesting that online platforms might act as a means to improve friendship quality (105). For this purpose, Gwynette and colleagues explored Facebook's therapeutic potential as a tool to improve social skills in adolescents with ASD. Their web-based intervention, according to the authors, could have the potential to facilitate interventions, leading to higher engagement with peers through the virtual environment (106). In the context of neurodevelopmental disorders, Asperger syndrome is characterized by significant difficulties in social interaction and non-verbal communication; as a consequence, they could be more vulnerable to cyberbullying victimization on online applications. Findings in the literature suggest that, although adolescents with Asperger syndrome use social media less than their peers, the percentage and frequency of cyberbullying are similar (107). Another neurodevelopmental condition is attention-deficit/hyperactivity disorder (ADHD), which is defined by persistent inattention, hyperactivity, and sometimes impulsivity. These features, combined with online-based platforms, might lead to addictive social media behaviors, with further consequences on mental health, productivity, and academic scores (48). Studies analyzing the correlation between ADHD traits and social media found that a large number of adolescents with ADHD own more than one Facebook account, showed greater overuse compared to their counterparts (39), and ADHD symptoms are positively associated with Facebook addictive use (48). Furthermore, teenagers with more marked ADHD traits were more likely to develop problematic usage of Internet-based services and less likely to remit from problematic Internet usage (108).

## 3. Gene-by-Environment Contribution to Understand Behavior on Social Media

The hypothesis that genetic features influence behavior and social interactions has been corroborated in several studies [for a review, see (109)], and so is the notion that human behavior and psychological traits are modulated by the interaction between genetic variation and environmental factors (21). Due to the intrinsic interactional nature of social media platforms, it is important to deepen the exploration of concurrent factors that could explain underlying mechanisms related to online interaction adopting integrated methodologies widely used for offline social behavior, that is, the gene-by-environment interaction framework. Few studies report results about genetic contribution in Internet-related usage. Two studies on Turkish twins on communication and social media reported that genetic and environmental effects were equally influential on problematic Internet usage especially in male twin-pairs (110). Another twin study highlighted the impact of genetics on mobile phone use (111). These results have been corroborated by a more recent investigation by York, who focused specifically on social media use (e.g., contact friends and contact family) even after controlling for demographic factors (112). A recent study by Deryakulu and Ursavaş examines the extent to which nomophobia can be explained by genetic and environmental factors, revealing that the dimensions which were more explained by genes were “losing connectedness” and “giving up convenience,” while environmental factors were more related to the fear of “not being able to communicate” and “not being able to access” (113). Familiar context represents a factor of great interest in shaping social behavior, especially at the developmental stage, and perception of parental warmth or intrusiveness can influence social media usage in adolescents. With regard to the genetic contribution within the frame of recalled parental bonding, a recent exploration found that people who are genetically more sensitive to environmental factors, represented by oxytocin receptor polymorphisms, with a history of perceived high maternal overprotection tend to show a higher social desirability index on Instagram (114). This index, which describes the ratio between the number of following and followed profiles, could be used for future studies to unveil some tendencies underlying user behavior on Instagram.

## 4. Social Media Usage and Neural Mechanisms

Evidence deriving from the neuroscientific field reveals a link between online social behaviors and regulation of neural mechanisms. A functional magnetic resonance imaging (fMRI) study conducted by Meshi and colleagues reports that social media engagement is linked to activity in the ventral striatum (vSTR) and adjoining structures of the nucleus accumbens (115). More precisely, the authors found an association between levels of activation of these areas and in response to social feedback identified that were relevant to participants' social reputation (a surrogate for “likes” on Facebook). Another study describes greater recruitment of the vSTR in relation to more popular shared pictures compared to less socially endorsed ones (116). As for structural evidence on gray matter volume and social media habits, the striatal region was found to be linked to daily smartphone checking (117) and heavy social media usage (118). Recent evidence also suggests the involvement of the right lateral orbitofrontal cortex, linking a decreased volume in that area with an excessive usage of social media sites (119). With regard to impulse control, reduced gray matter volume in the anterior cingulate cortex was found in people with high tendencies in developing an addictive attitude toward instant messaging services (120) and “multitasking" users, suggesting that social media usage is highly involved in the control of inhibitory mechanisms (121). Another relevant study by Moisala and colleagues on media multitasking showed increased activity in the right side of the prefrontal cortex while participants were subjected to a cognitive task; this result was explained by the authors as a reflection of mental struggle in recruiting resources in executive control (122). With regard to social cognition in adolescence, fMRI studies found that online rejection by peers or other users elicits an increased activity in the medial prefrontal cortex, which is strongly associated with offline rejection (20), and elicits neural responses in the dorsal anterior cingulate cortex, the subgenual anterior cingulate cortex, and the anterior insula, which are areas generally linked to “social pain” (20, 123) and depression (124). The immediate and long-term effects of frequent and prolonged social media usage on neural structures and activity have yet to be elucidated.

## 5. Conclusion

In just one decade, individuals' lives and their social behavior have been tremendously changed by the phenomenon of social media. Emerging technologies and platforms provide users with a wide range of activities, leisure, and the possibility to interact with friends, families, or strangers. Although different patterns of usage are moderated by a set of individual features concerning genetic, environment, temperament, and personal needs, it is undeniable that online social media have become an integrated part of people's daily lives. This leads to the necessity, in research fields linked to human behavior, to understand if, how, and to what extent these platforms are modifying our brain mechanisms, interactions, and the concept of well-being. During developmental stages, such as adolescence and early adulthood, several changes occur not only with regard to neural functions but also in social patterns, as young people have increasing opportunity to test themselves as individuals in more autonomous social interactions. As for social media, the most popular platforms require users to be at least 13 years old to own a profile and have access to the services. Although this limit is easily bypassed, it is difficult to have a clear overview of the sociodemographic information of young users and of different patterns of usage or effects of social media in early adolescence (10–14), middle adolescence (15–17), and young adulthood (18–21), as the early adolescence population should not be able to access and be engaged in virtual interactions on such platforms. This issue rebounds in a lack of studies considering this distinction, representing a further challenge to future research. Lots of efforts have been invested in creating new tools for assessing people's attitudes toward social media usage, such as the creation and validation of new scales (84, 125–129) and to interpret results within a fitting theoretical frame. Social media provide unprecedented opportunities to trace online activity and to keep track of interaction dynamics at different stages. This allows researchers to overcome issues related to self-report questionnaires and to benefit from leveraging real-time data over time more easily. Specifically, the increasing utilization of hashtags might help in detecting and monitoring targeted topics or risky behaviors, despite the risk of misappropriate use of words (for instance, sometimes people refer to “anxiety" or “depression" when perceiving alterations in preoccupation or mood but with these not being of clinical interest or diagnosed. For sure, the use of hashtags is a powerful tool to build communities and support people's journey to recover, to witness, to join a cause, or to increase awareness around a specific topic related to mental health. As the number of social media applications increases, with each having its own specific features, there is a need to separate problematic behaviors or effects according to the platforms. In fact, since the advent of social networks sites, multiple platforms have succeeded one another, gaining immediate popularity. Some of them are not used anymore, such as Google+, or had a drastic loss of users, like MySpace. Lately, a new social network site named TikTok, formerly known as Musical.ly, has risen especially among the youth, changing to some extent the way social media are used. From the simple sharing of text, music, or pictures, social media has rapidly evolved, becoming more dynamic and providing the possibility to get immediate and abundant feedback, to join wide online communities based on common interests, and to involve users' talents or attitudes with so-called “challenges." Although TikTok had gained terrific popularity in the course of the last year, no studies regarding the potential outcomes deriving from a problematic usage are available. As social media sites are quickly developing, research appears to struggle in keeping pace with not only the new online functionalities but also with ways of interactions among users that, in turn, might alter parameters in longitudinal studies, like the amount of time spent online. This is partially due to the fact that effects can be explored in terms of both a short and long term, each with different consequences. Moreover, the social media platforms resent of the users' preferences and mass tendencies, and what is new and trendy today might swiftly lose people's interest (130). Since keeping track of how communication technologies evolve across the years can be a precious resource on developmental trajectories, it becomes of great importance for researchers to build and rely on constantly updated evidence. The creation and rise of new technologies has resulted in new behaviors and, consequently, new names for these behaviors. Neologisms like “*nomophobia*,” “*selfie*,” “*phubbing*,” “*FoMO*,” and “*vaguebooking*” have appeared for some years, defining specific behaviors or state of minds, that need further analysis, as they represent new, unexplored facets of human behavior. Research in psychological fields would also benefit from the exploration of specific types of interaction, such as the creation of multiple accounts, the fruition of live streaming video services, and behaviors like un-tagging people from posts or pictures or unfollowing/unfriending people in order to better understand the effects of mechanisms related to virtual social inclusion or exclusion. Although social media allows for greater ease of recruitment and testing of a greater number of participants in more efficient ways—sometimes comparable to laboratory testing sessions (131)—a lack of knowledge still persists regarding the involvement of specific brain regions or genetic susceptibility in developing a certain social media-related disorder. In addition, only a few studies adopted a longitudinal design, while most of the evidence is still based on a cross-sectional methodology that does not fully allow researchers to study in detail the direction of the association between social media usage and psychological well-being. Furthermore, the mental health community should commit to find a solution in considering social media-related issues as being separate from other forms of problematic online behaviors or usage. As there is no separated diagnosis, social media concerns are often included or subsumed within the Internet addiction frame, leading to an incorrect framing of the problem, especially with regard to the social connotation that primarily describes and defines these kinds of services. New evidence in these fields would be of great support for practitioners in a twofold way: on the one hand, information shared on social media sites and patterns of usage of new technologies could be implemented in clinical work for both a more complete assessment, and, on the other hand, it would be possible to profile more user-based interventions merging both online and offline strategies.

## Author Contributions

IC, GE, and BL conceived the paper. IC performed the search, interpreted the literature, and wrote the paper. GE, MN, and BL reviewed and edited the paper. GE submitted the paper. All the authors reviewed the final version of the paper before submission.

## Conflict of Interest

The authors declare that the research was conducted in the absence of any commercial or financial relationships that could be construed as a potential conflict of interest.

## References

[1] Greenfield P Yan Z. Children, adolescents, and the Internet: a new field of inquiry in developmental psychology. Dev Psychol. (2006) 42:391. 10.1037/0012-1649.42.3.391 16756431

[2] Lloyd A Social media, help or hindrance: what role does social media play in young people's mental health. Psychiatr Danub. (2014) 26(Suppl. 1):340–6. 25413562

[3] Radovic A Gmelin T Stein BD Miller E. Depressed adolescents' positive and negative use of social media. J Adolesc. (2017) 55:5–15. 10.1016/j.adolescence.2016.12.002 27997851 PMC5485251

[4] Lieberman A Schroeder J. Two social lives: how differences between online and offline interaction influence social outcomes. Curr Opin Psychol. (2020) 31:16–21. 10.1016/j.copsyc.2019.06.022 31386968

[5] Khan S Gagné M Yang L Shapka J Exploring the relationship between adolescents' self-concept and their offline and online social worlds. Comput Hum Behav. (2016) 55:940–5. 10.1016/j.chb.2015.09.046

[6] Warburton S Digital identity and social media. Hershey, PA: IGI Global (2012).

[7] Sa'ed HZ Sweileh WM Awang R Al-Jabi SW. Global trends in research related to social media in psychology: mapping and bibliometric analysis. Int J Ment Health Syst. (2018) 12:4. 10.1186/s13033-018-0182-6 29387147 PMC5775539

[8] Bányai F Zsila Á Király O Maraz A Elekes Z Griffiths MD. Problematic social media use: results from a large-scale nationally representative adolescent sample. PLoS ONE. (2017) 12:e0169839. 10.1371/journal.pone.0169839 28068404 PMC5222338

[9] Twenge JM More time on technology, less happiness? Associations between digital-media use and psychological well-being. Curr Dir Psychol Sci. (2019). 28:372–9. 10.1177/0963721419838244

[10] Viner RM Aswathikutty-Gireesh A Stiglic N Hudson LD Goddings AL Ward JL. Roles of cyberbullying, sleep, and physical activity in mediating the effects of social media use on mental health and wellbeing among young people in England: a secondary analysis of longitudinal data. Lancet Child Adolesc Health. (2019) 3:685–96. 10.1016/S2352-4642(19)30186-5 31420213

[11] Tran BX Hinh ND Nguyen LH Le BN Nong VM Thuc VTM. A study on the influence of internet addiction and online interpersonal influences on health-related quality of life in young Vietnamese. BMC Public Health. (2017) 17:138. 10.1186/s12889-016-3983-z 28143462 PMC5282902

[12] Kuss DJ Griffiths MD Excessive online social networking: can adolescents become addicted to Facebook? Educ Health. (2011) 29:63–6.

[13] Valkenburg PM Peter J Adolescents' identity experiments on the Internet: consequences for social competence and self-concept unity. Commun Res. (2008) 35:208–31. 10.1177/0093650207313164

[14] Kirschner PA Karpinski AC Facebook® and academic performance. Comput Hum Behav. (2010) 26:1237–45. 10.1016/j.chb.2010.03.024

[15] Reinecke L Meier A Beutel ME Schemer C Stark B Wölfling K. The relationship between trait procrastination, internet use, and psychological functioning: results from a community sample of German adolescents. Front Psychol. (2018) 9:913. 10.3389/fpsyg.2018.00913 29942268 PMC6004405

[16] Hussain Z Griffiths MD. Problematic social networking site use and comorbid psychiatric disorders: a systematic review of recent large-scale studies. Front Psychiatry. (2018) 9:686. 10.3389/fpsyt.2018.00686 30618866 PMC6302102

[17] Keles B McCrae N Grealish A A systematic review: the influence of social media on depression, anxiety and psychological distress in adolescents. Int J Adolesc Youth. (2020) 25:79–93. 10.1080/02673843.2019.1590851

[18] Abi-Jaoude E Naylor KT Pignatiello A Smartphones, social media use and youth mental health. CMAJ. (2020) 192:E136–41. 10.1503/cmaj.190434 32041697 PMC7012622

[19] Paulus MP Squeglia LM Bagot K Jacobus J Kuplicki R Breslin FJ. Screen media activity and brain structure in youth: evidence for diverse structural correlation networks from the ABCD study. Neuroimage. (2019) 185:140–53. 10.1016/j.neuroimage.2018.10.040 30339913 PMC6487868

[20] Crone EA Konijn EA. Media use and brain development during adolescence. Nat Commun. (2018) 9:588. 10.1530/ey.15.7.4 29467362 PMC5821838

[21] Cataldo I Azhari A Lepri B Esposito G. Oxytocin receptors (OXTR) and early parental care: an interaction that modulates psychiatric disorders. Res Dev Disabil. (2018) 82:27–38. 10.1016/j.ridd.2017.10.007 29033100

[22] Feldman R Monakhov M Pratt M Ebstein RP. Oxytocin pathway genes: evolutionary ancient system impacting on human affiliation, sociality, and psychopathology. Biol Psychiatry. (2016) 79:174–84. 10.1016/j.biopsych.2015.08.008 26392129

[23] Szwedo DE Mikami AY Allen JP. Qualities of peer relations on social networking websites: predictions from negative mother–teen interactions. J Res Adolesc. (2011) 21:595–607. 10.1111/j.1532-7795.2010.00692.x 21860584 PMC3158584

[24] Moreno MA Kacvinsky L Pumper M Wachowski L Whitehill JM. Associations between social media displays and event-specific alcohol consumption by college students. WMJ. (2013) 112:251. 24511865 PMC3929207

[25] Pumper MA Moreno MA. Identifying high-risk alcohol users in first-year college students: attitude, intention, and Facebook. J Alcohol Drug Depend. (2013) 1:1000128. 10.4172/2329-6488.1000128 25419542 PMC4238920

[26] Tiggemann M Slater A. NetGirls: the Internet, Facebook, and body image concern in adolescent girls. Int J Eat Disord. (2013) 46:630–3. 10.1002/eat.22141 23712456

[27] D'Angelo J Kerr B Moreno MA. Facebook displays as predictors of binge drinking: from the virtual to the visceral. Bull Sci Technol Soc. (2014) 34:159–69. 10.1177/0270467615584044 26412923 PMC4581523

[28] Huang GC Unger JB Soto D Fujimoto K Pentz MA Jordan-Marsh M. Peer influences: the impact of online and offline friendship networks on adolescent smoking and alcohol use. J Adolesc Health. (2014) 54:508–14. 10.1016/j.jadohealth.2013.07.001 24012065 PMC4694047

[29] Birnbaum ML Rizvi AF Correll CU Kane JM Confino J. Role of social media and the I nternet in pathways to care for adolescents and young adults with psychotic disorders and non-psychotic mood disorders. Early Interv Psychiatry. (2017) 11:290–5. 10.1111/eip.12237 25808317 PMC4580496

[30] Nesi J Rothenberg WA Hussong AM Jackson KM. Friends' alcohol-related social networking site activity predicts escalations in adolescent drinking: mediation by peer norms. J Adolesc Health. (2017) 60:641–7. 10.1016/j.jadohealth.2017.01.009 28325545 PMC6402495

[31] Bert F Gualano MR Camussi E Siliquini R. Risks and threats of social media websites: twitter and the Proana movement. Cyberpsychol Behav Soc Netw. (2016) 19:233–8. 10.1089/cyber.2015.0553 26991868

[32] Frison E Eggermont S Exploring the relationships between different types of Facebook use, perceived online social support, and adolescents' depressed mood. Soc Sci Comput Rev. (2016) 34:153–71. 10.1177/0894439314567449

[33] Marczinski CA Hertzenberg H Goddard P Maloney SF Stamates AL O'Connor K. Alcohol-related Facebook activity predicts alcohol use patterns in college students. Addict Res Theor. (2016) 24:398–405. 10.3109/16066359.2016.1146709 28138317 PMC5271575

[34] Moreno MA Arseniev-Koehler A Litt D Christakis D. Evaluating college students' displayed alcohol references on facebook and twitter. J Adolesc Health. (2016) 58:527–32. 10.1016/j.jadohealth.2016.01.005 26995291 PMC5942193

[35] Naeemi S Tamam E The relationship between emotional dependence on facebook and psychological well-being in adolescents aged 13–16. Child Indic Res. (2017) 10:1095–106. 10.1007/s12187-016-9438-3

[36] Sampasa-Kanyinga H Chaput JP. Use of social networking sites and alcohol consumption among adolescents. Public Health. (2016) 139:88–95. 10.1016/j.puhe.2016.05.005 27311992

[37] Abar CC Farnett S Mendola K Koban K Sarra S Relationships between parent–child social media interactions and health behaviors. J Subst Use. (2018) 23:335–7. 10.1080/14659891.2017.1410586

[38] Frison E Eggermont S. Browsing, posting, and liking on Instagram: the reciprocal relationships between different types of Instagram use and adolescents' depressed mood. Cyberpsychol Behav Soc Netw. (2017) 20:603–9. 10.1089/cyber.2017.0156 29039700

[39] Gul H Yurumez Solmaz E Gul A Oner O Facebook overuse and addiction among Turkish adolescents: are ADHD and ADHD-related problems risk factors? Psychiatry Clin Psychopharmacol. (2018) 28:80–90. 10.1080/24750573.2017.1383706

[40] Jacob N Evans R Scourfield J. The influence of online images on self-harm: a qualitative study of young people aged 16–24. J Adolesc. (2017) 60:140–7. 10.1016/j.adolescence.2017.08.001 28881214 PMC5614108

[41] Pontes HM. Investigating the differential effects of social networking site addiction and Internet gaming disorder on psychological health. J Behav Addict. (2017) 6:601–10. 10.1556/2006.6.2017.075 29130329 PMC6034963

[42] Spilkova J Chomynova P Csemy L. Predictors of excessive use of social media and excessive online gaming in Czech teenagers. J Behav Addict. (2017) 6:611–9. 10.1556/2006.6.2017.064 29039223 PMC6034940

[43] Van Rooij AJ Ferguson CJ Van de Mheen D Schoenmakers TM Time to abandon Internet Addiction? Predicting problematic Internet, game, and social media use from psychosocial well-being and application use. Clin Neuropsychiatry. (2017) 14:113–21.

[44] Weinstein E Adolescents' differential responses to social media browsing: exploring causes and consequences for intervention. Comput Hum Behav. (2017) 76:396–405. 10.1016/j.chb.2017.07.038

[45] Brown RC Fischer T Goldwich AD Keller F Young R Plener PL # cutting: non-suicidal self-injury (NSSI) on Instagram. Psychol Med. (2018) 48:337–46. 10.1017/S0033291717001751 28705261

[46] Muzaffar N Brito EB Fogel J Fagan D Kumar K Verma R. The association of adolescent Facebook behaviours with symptoms of social anxiety, generalized anxiety, and depression. J Can Acad Child Adolesc Psychiatry. (2018) 27:252. 30487941 PMC6254262

[47] Niu GF Luo YJ Sun XJ Zhou ZK Yu F Yang SL. Qzone use and depression among Chinese adolescents: a moderated mediation model. J Affect Disord. (2018) 231:58–62. 10.1016/j.jad.2018.01.013 29453010

[48] Settanni M Marengo D Fabris MA Longobardi C The interplay between ADHD symptoms and time perspective in addictive social media use: a study on adolescent Facebook users. Child Youth Serv Rev. (2018) 89:165–70. 10.1016/j.childyouth.2018.04.031

[49] Chang L Li P Loh RSM Chua THH. A study of Singapore adolescent girls' selfie practices, peer appearance comparisons, and body esteem on Instagram. Body Image. (2019) 29:90–9. 10.1016/j.bodyim.2019.03.005 30884385

[50] de Vries DA Vossen HGM van der Kolk-van der Boom P. Social media and body dissatisfaction: investigating the attenuating role of positive parent–Adolescent relationships. J Youth Adolesc. (2019) 48:527–36. 10.1007/s10964-018-0956-9 30478819 PMC6394528

[51] Louragli I Ahami A Khadmaoui A Aboussaleh Y Lamrani AC Behavioral analysis of adolescent's students addicted to facebook and its impact on performance and mental health. Acta Neuropsychol. (2019) 17:427–39. 10.5604/01.3001.0013.6550

[52] Negriff S. Depressive symptoms predict characteristics of online social networks. J Adolesc Health. (2019) 65:101–6. 10.1016/j.jadohealth.2019.01.026 30956137 PMC6589394

[53] Przepiorka A Blachnio A. The role of Facebook intrusion, depression, and future time perspective in sleep problems among adolescents. J Res Adolesc. (2019) 30:559–69. 10.1111/jora.12543 31868972

[54] Raudsepp L Kais K. Longitudinal associations between problematic social media use and depressive symptoms in adolescent girls. Prev Med Rep. (2019) 15:100925. 10.1016/j.pmedr.2019.100925 31304081 PMC6603436

[55] Savolainen I Oksanen A Kaakinen M Sirola A Miller BL Paek HJ. The Association between social media use and hazardous alcohol use among youths: a four-country study. Alcohol Alcohol. (2020) 55:86–95. 10.1093/alcalc/agz088 31761930

[56] Steers MLN Neighbors C Wickham RE Petit WE Kerr B Moreno MA. My friends, I'm# SOTALLYTOBER: A longitudinal examination of college students' drinking, friends' approval of drinking, and Facebook alcohol-related posts. Digit Health. (2019) 5:2055207619845449. 10.1177/2055207619845449 31105968 PMC6505233

[57] Vannucci A Ohannessian CM. Social media use subgroups differentially predict psychosocial well-being during early adolescence. J Youth Adolesc. (2019) 48:1469–93. 10.1007/s10964-019-01060-9 31256313

[58] Yurdagül C Kircaburun K Emirtekin E Wang P Griffiths MD Psychopathological consequences related to problematic instagram use among adolescents: the mediating role of body image dissatisfaction and moderating role of gender. Int J Ment Health Addict. (2019) 1–13. 10.1007/s11469-019-00071-8

[59] Boursier V Gioia F Griffiths MD. Objectified body consciousness, body image control in photos, and problematic social networking: the role of appearance control beliefs. Front Psychol. (2020) 11:147. 10.3389/fpsyg.2020.00147 32158409 PMC7052303

[60] Fardouly J Magson NR Rapee RM Johnco CJ Oar EL. The use of social media by Australian preadolescents and its links with mental health. J Clin Psychol. (2020) 76:1304–26. 10.1002/jclp.22936 32003901

[61] Stockdale LA Coyne SM. Bored and online: reasons for using social media, problematic social networking site use, and behavioral outcomes across the transition from adolescence to emerging adulthood. J Adolesc. (2020) 79:173–83. 10.1016/j.adolescence.2020.01.010 31978836

[62] Brown RC Fischer T Goldwich DA Plener PL. “I just finally wanted to belong somewhere”—Qualitative analysis of experiences with posting pictures of self-injury on instagram. Front Psychiatry. (2020) 11:274. 10.3389/fpsyt.2020.00274 32372983 PMC7186324

[63] Danaux X Gandy D FontAwesome. (2016). Available online at: http://mirror.kumi.systems/ctan/fonts/fontawesome/doc/fontawesome.pdf

[64] Lenhard W Lenhard A Calculation of Effect Sizes. Dettelbach (2016).

[65] American Psychiatric Pub Diagnostic and Statistical Manual of Mental Disorders (DSM-5®). Washington, DC: American Psychiatric Pub (2013).

[66] Nesi J Prinstein MJ. Using social media for social comparison and feedback-seeking: gender and popularity moderate associations with depressive symptoms. J Abnorm Child Psychol. (2015) 43:1427–38. 10.1007/s10802-015-0020-0 25899879 PMC5985443

[67] Twenge JM Cooper AB Joiner TE Duffy ME Binau SG. Age, period, and cohort trends in mood disorder indicators and suicide-related outcomes in a nationally representative dataset, 2005–2017. J Abnorm Psychol. (2019) 128:185–99. 10.1037/abn0000410 30869927

[68] Aalbers G McNally RJ Heeren A Wit SD Fried EI. Social media and depression symptoms: a network perspective. J Exp Psychol Gen. (2018) 148:1454–62. 10.1037/xge0000528 30507215

[69] Brooks S Longstreet P Social networking's peril: cognitive absorption, social networking usage, and depression. Cyberpsychology. (2015) 9:21–39. 10.5817/CP2015-4-5

[70] Tobin SJ Vanman EJ Verreynne M Saeri AK Threats to belonging on Facebook: lurking and ostracism. Soc Influence. (2015) 10:31–42. 10.1080/15534510.2014.893924

[71] Kross E Verduyn P Demiralp E Park J Lee DS Lin N. Facebook use predicts declines in subjective well-being in young adults. PLoS ONE. (2013) 8:e69841. 10.1371/journal.pone.0069841 23967061 PMC3743827

[72] Ehrenreich SE Underwood MK. Adolescents' internalizing symptoms as predictors of the content of their Facebook communication and responses received from peers. Transl Issues Psychol Sci. (2016) 2:227. 10.1037/tps0000077 28083544 PMC5222594

[73] Harter S The Construction of the Self: Developmental and Sociocultural Foundations. New York, NY: Guilford Publications (2015).

[74] Chou HTG Edge N. “They are happier and having better lives than I am”: the impact of using Facebook on perceptions of others' lives. Cyberpsychol Behav Soc Netw. (2012) 15:117–21. 10.1089/cyber.2011.0324 22165917

[75] Davila J Hershenberg R Feinstein BA Gorman K Bhatia V Starr LR. Frequency and quality of social networking among young adults: associations with depressive symptoms, rumination, and corumination. Psychol Popul Media Cult. (2012) 1:72. 10.1037/a0027512 24490122 PMC3907111

[76] Steers MLN Wickham RE Acitelli LK Seeing everyone else's highlight reels: how Facebook usage is linked to depressive symptoms. J Soc Clin Psychol. (2014) 33:701–31. 10.1521/jscp.2014.33.8.701

[77] Appel H Gerlach AL Crusius J The interplay between Facebook use, social comparison, envy, and depression. Curr Opin Psychol. (2016) 9:44–9. 10.1016/j.copsyc.2015.10.006

[78] Bollen J Gonçalves B van de Leemput I Ruan G The happiness paradox: your friends are happier than you. EPJ Data Sci. (2017) 6:4 10.1140/epjds/s13688-017-0100-1

[79] Frison E Subrahmanyam K Eggermont S. The short-term longitudinal and reciprocal relations between peer victimization on Facebook and adolescents' well-being. J Youth Adolesc. (2016) 45:1755–71. 10.1007/s10964-016-0436-z 26880284

[80] McCloskey W Iwanicki S Lauterbach D Giammittorio DM Maxwell K. Are Facebook “friends” helpful? Development of a Facebook-based measure of social support and examination of relationships among depression, quality of life, and social support. Cyberpsychol Behav Soc Netw. (2015) 18:499–505. 10.1089/cyber.2014.0538 26348809

[81] Hoge E Bickham D Cantor J. Digital media, anxiety, and depression in children. Pediatrics. (2017) 140(Suppl. 2):S76–80. 10.1542/peds.2016-1758G 29093037

[82] Calancie O Ewing L Narducci LD Horgan S Khalid-Khan S Exploring how social networking sites impact youth with anxiety: a qualitative study of Facebook stressors among adolescents with an anxiety disorder diagnosis. Cyberpsychology. (2017) 11 10.5817/CP2017-4-2

[83] Oberst U Wegmann E Stodt B Brand M Chamarro A. Negative consequences from heavy social networking in adolescents: the mediating role of fear of missing out. J Adolesc. (2017) 55:51–60. 10.1016/j.adolescence.2016.12.008 28033503

[84] Przybylski AK Murayama K DeHaan CR Gladwell V Motivational, emotional, and behavioral correlates of fear of missing out. Comput Hum Behav. (2013) 29:1841–8. 10.1016/j.chb.2013.02.014

[85] Andreassen CS Online social network site addiction: a comprehensive review. Curr Addict Rep. (2015) 2:175–84. 10.1007/s40429-015-0056-9

[86] Dhir A Yossatorn Y Kaur P Chen S Online social media fatigue and psychological wellbeing—A study of compulsive use, fear of missing out, fatigue, anxiety and depression. Int J Inform Manage. (2018) 40:141–52. 10.1016/j.ijinfomgt.2018.01.012

[87] Dempsey AE O'Brien KD Tiamiyu MF Elhai JD. Fear of missing out (FoMO) and rumination mediate relations between social anxiety and problematic Facebook use. Addict Behav Rep. (2019) 9:100150. 10.1016/j.abrep.2018.100150 31193746 PMC6542373

[88] Beyens I Frison E Eggermont S “I don't want to miss a thing”: Adolescents' fear of missing out and its relationship to adolescents' social needs, Facebook use, and Facebook related stress. Comput Hum Behav. (2016) 64:1–8. 10.1016/j.chb.2016.05.083

[89] McLean SA Jarman HK Rodgers RF. How do “selfies” impact adolescents' well-being and body confidence? A narrative review. Psychol Res Behav Manage. (2019) 12:513. 10.2147/PRBM.S177834 31372071 PMC6628890

[90] Forrest KY Stuhldreher WL Patterns and correlates of body image dissatisfaction and distortion among college students. Am J Health Stud. (2007) 22:18–25.

[91] Pater JA Haimson OL Andalibi N Mynatt ED “Hunger Hurts but Starving Works” Characterizing the Presentation of Eating Disorders Online. In: Proceedings of the 19th ACM Conference on Computer-Supported Cooperative Work & Social Computing. San Francisco, CA (2016). p. 1185–200.

[92] Curtis BL Lookatch SJ Ramo DE McKay JR Feinn RS Kranzler HR. Meta-analysis of the association of alcohol-related social media use with alcohol consumption and alcohol-related problems in adolescents and young adults. Alcohol Clin Exp Res. (2018) 42:978–86. 10.1111/acer.13642 29786874 PMC5984178

[93] Westgate EC Neighbors C Heppner H Jahn S Lindgren KP. “I will take a shot for every ‘like'I get on this status”: posting alcohol-related Facebook content is linked to drinking outcomes. J Stud Alcohol Drugs. (2014) 75:390–8. 10.15288/jsad.2014.75.390 24766750 PMC4002853

[94] Marchant A Hawton K Stewart A Montgomery P Singaravelu V Lloyd K. A systematic review of the relationship between internet use, self-harm and suicidal behaviour in young people: the good, the bad and the unknown. PLoS ONE. (2017) 12:e0181722. 10.1371/journal.pone.0181722 28813437 PMC5558917

[95] Cash SJ Thelwall M Peck SN Ferrell JZ Bridge JA. Adolescent suicide statements on MySpace. Cyberpsychol Behav Soc Netw. (2013) 16:166–74. 10.1089/cyber.2012.0098 23374167

[96] Brown RC Bendig E Fischer T Goldwich AD Baumeister H Plener PL. Can acute suicidality be predicted by Instagram data? Results from qualitative and quantitative language analyses. PLoS ONE. (2019) 14:e0220623. 10.1371/journal.pone.0220623 31504042 PMC6736249

[97] Miguel EM Chou T Golik A Cornacchio D Sanchez AL DeSerisy M. Examining the scope and patterns of deliberate self-injurious cutting content in popular social media. Depress Anxiety. (2017) 34:786–93. 10.1002/da.22668 28661053

[98] Moreno MA Ton A Selkie E Evans Y. Secret society 123: understanding the language of self-harm on Instagram. J Adolesc Health. (2016) 58:78–84. 10.1016/j.jadohealth.2015.09.015 26707231 PMC5322804

[99] Luxton DD June JD Fairall JM. Social media and suicide: a public health perspective. Am J Public Health. (2012) 102:195–200. 10.2105/AJPH.2011.300608 22401525 PMC3477910

[100] Pettalia JL Levin E Dickinson J Cyberbullying: eliciting harm without consequence. Comput Hum. Behav. (2013) 29:2758–65. 10.1016/j.chb.2013.07.020

[101] Barlett CP Gentile DA Chng G Li D Chamberlin K Social media use and cyberbullying perpetration: a longitudinal analysis. Violence Gender. (2018) 5:191–7. 10.1089/vio.2017.0047

[102] Shakir T. Bhandari N. Andrews A. Zmitrovich A. McCracken C. Gadomski J. (2019). Do our adolescents know they are cyberbullying victims? J. Infant Child Adolesc. Psychother. 18, 93–101. 10.1080/15289168.2018.1565004

[103] Chatzakou D Leontiadis I Blackburn J De Cristofaro E Stringhini G Vakali A Detecting cyberbullying and cyberaggression in social media. arXiv preprint arXiv:190708873 (2019). 10.1145/3343484

[104] Plemmons G Hall M Doupnik S Gay J Brown C Browning W Hospitalization for suicide ideation or attempt: 2008–2015. Pediatrics. (2018) 141:e20172426 10.1542/peds.2017-2426 29769243

[105] van Schalkwyk GI Marin CE Ortiz M Rolison M Qayyum Z McPartland JC. Social media use, friendship quality, and the moderating role of anxiety in adolescents with autism spectrum disorder. J Autism Dev Disord. (2017) 47:2805–13. 10.1007/s10803-017-3201-6 28616856 PMC6688174

[106] Gwynette MF Morriss D Warren N Truelove J Warthen J Ross CP. Social skills training for adolescents with autism spectrum disorder using facebook (project rex connect): a survey study. JMIR Ment Health. (2017) 4:e4. 10.2196/mental.6605 28115297 PMC5294368

[107] Iglesias OB Sanchez LEG Rodríguez MÁA Do young people with Asperger syndrome or intellectual disability use social media and are they cyberbullied or cyberbullies in the same way as their peers? Psicothema. (2019) 31:30–7. 30664408 10.7334/psicothema2018.243

[108] Choi BY Huh S Kim DJ Suh SW Lee SK Potenza MN. Transitions in problematic internet use: a one-year longitudinal study of boys. Psychiatry Invest. (2019) 16:433–42. 10.30773/pi.2019.04.02.1 31247702 PMC6603706

[109] Ebstein RP Israel S Chew SH Zhong S Knafo A. Genetics of human social behavior. Neuron. (2010) 65:831–44. 10.1016/j.neuron.2010.02.020 20346758

[110] Deryakulu D Ursavaş ÖF Genetic and environmental influences on problematic Internet use: a twin study. Comput Hum Behav. (2014) 39:331–8. 10.1016/j.chb.2014.07.038

[111] Miller G Zhu G Wright MJ Hansell NK Martin NG. The heritability and genetic correlates of mobile phone use: a twin study of consumer behavior. Twin Res Hum Genet. (2012) 15:97–106. 10.1375/twin.15.1.97 22784459

[112] York C A regression approach to testing genetic influence on communication behavior: social media use as an example. Comput Hum Behav. (2017) 73:100–9. 10.1016/j.chb.2017.03.029

[113] Deryakulu D Ursavaş ÖF Genetic and environmental sources of nomophobia: a small-scale Turkish Twin study. Addicta. (2019) 6:147–62. 10.15805/addicta.2019.6.1.0028

[114] Bonassi A Cataldo I Gabrieli G Foo JN Lepri B Esposito G. Oxytocin Receptor Gene polymorphism and early parental bonding interact in shaping Instagram social behaviour. PsyArXiv. (2019). Available online at: psyarxiv.com/n6mgv. 10.3390/ijerph17197232 PMC7579356 33022913

[115] Meshi D Morawetz C Heekeren HR. Nucleus accumbens response to gains in reputation for the self relative to gains for others predicts social media use. Front Hum Neurosci. (2013) 7:439. 10.3389/fnhum.2013.00439 24009567 PMC3757324

[116] Sherman LE Payton AA Hernandez LM Greenfield PM Dapretto M. The power of the like in adolescence: effects of peer influence on neural and behavioral responses to social media. Psychol Sci. (2016) 27:1027–35. 10.1177/0956797616645673 27247125 PMC5387999

[117] Montag C Markowetz A Blaszkiewicz K Andone I Lachmann B Sariyska R. Facebook usage on smartphones and gray matter volume of the nucleus accumbens. Behav Brain Res. (2017) 329:221–8. 10.1016/j.bbr.2017.04.035 28442353

[118] He Q Turel O Brevers D Bechara A. Excess social media use in normal populations is associated with amygdala-striatal but not with prefrontal morphology. Psychiatry Res Neuroimaging. (2017) 269:31–5. 10.1016/j.pscychresns.2017.09.003 28918269

[119] Lee D Namkoong K Lee J Lee BO Jung YC. Lateral orbitofrontal gray matter abnormalities in subjects with problematic smartphone use. J Behav Addict. (2019) 8:404–11. 10.1556/2006.8.2019.50 31545101 PMC7044619

[120] Montag C Zhao Z Sindermann C Xu L Fu M Li J. Internet Communication Disorder and the structure of the human brain: initial insights on WeChat addiction. Sci Rep. (2018) 8:2155. 10.1038/s41598-018-19904-y 29391461 PMC5794793

[121] Loh KK Kanai R. Higher media multi-tasking activity is associated with smaller gray-matter density in the anterior cingulate cortex. PLoS ONE. (2014) 9:e106698. 10.1371/journal.pone.0106698 25250778 PMC4174517

[122] Moisala M Salmela V Hietajärvi L Salo E Carlson S Salonen O. Media multitasking is associated with distractibility and increased prefrontal activity in adolescents and young adults. Neuroimage. (2016) 134:113–21. 10.1016/j.neuroimage.2016.04.011 27063068

[123] Bayer JB O'Donnell MB Cascio CN Falk EB. Brain sensitivity to exclusion is associated with core network closure. Sci Rep. (2018) 8:16037. 10.1038/s41598-018-33624-3 30375417 PMC6207694

[124] Pandya M Altinay M Malone DA Anand A. Where in the brain is depression? Curr Psychiatry Rep. (2012) 14:634–42. 10.1007/s11920-012-0322-7 23055003 PMC3619732

[125] Andreassen CS Torsheim T Brunborg GS Pallesen S. Development of a Facebook addiction scale. Psychol Rep. (2012) 110:501–17. 10.2466/02.09.18.PR0.110.2.501-517 22662404

[126] Van den Eijnden R Lemmens J Valkenburg P The social media disorder scale: validity and psychometric properties. J Behav Addict. (2016) 5:13 10.1037/t53816-000

[127] Nick EA Cole DA Cho SJ Smith DK Carter TG Zelkowitz RL. The Online Social Support Scale: measure development and validation. Psychol Assess. (2018) 30:1127. 10.1037/pas0000558 29781664 PMC6107390

[128] Kwon M Kim DJ Cho H Yang S. The smartphone addiction scale: development and validation of a short version for adolescents. PLoS ONE. (2013) 8:e83558. 10.1371/journal.pone.0083558 24391787 PMC3877074

[129] Landoll RR La Greca AM Lai BS. Aversive Peer Experiences on Social Networking Sites: development of the Social Networking-Peer Experiences Questionnaire (SN-PEQ). J Res Adolesc. (2013) 23:695–705. 10.1111/jora.12022 24288449 PMC3839674

[130] Anderson KE Getting acquainted with social networks and apps: it is time to talk about TikTok. Library Hi Tech News. (2020).

[131] Casler K Bickel L Hackett E Separate but equal? A comparison of participants and data gathered via Amazon's MTurk, social media, and face-to-face behavioral testing. Comput Hum Behav. (2013) 29:2156–60. 10.1016/j.chb.2013.05.009

